# Supplementary material for: Comparison of 1-year mortality predictions from vendor-supplied versus academic model for cancer patients
Source: PeerJ. 2025 Feb 11;13:e18958. doi: 10.7717/peerj.18958 (PMC11827575; doi:10.7717/peerj.18958)
Supplement: Supplemental Information 2 [file peerj-13-18958-s002.docx]

**Supplemental Results**

Supplemental Table 1. One-year mortality prediction results including all patients, including those lost to follow-up prior to 1 year (n=1,399). For this version of the analysis, patients lost to follow-up prior to 1 year were counted as alive.

|  | Epic EOLCI | Stanford model |
| --- | --- | --- |
| AUC | 0.72 (0.69-0.75) | 0.80 (0.77-0.83) |
| Positive predictive value for high-risk group | 0.35 (n=537) | 0.40 (n=551) |
| Sensitivity for high-risk group | 0.63 | 0.75 |

Gini Index for EOLCI and Stanford model

We calculated the Gini index for both models, using the binarized model outputs (0 or 1 depending on whether the patient’s risk score was higher than that model’s high-risk threshold as discussed in the Results section). The Gini index was calculated by multiplying the proportion of high-risk predictions by the proportion of low-risk predictions, then multiplying by the sum of the positive predictive value (PPV) and negative predictive value (NPV) minus one, and dividing by the one-year mortality rate and its complement.

The Gini index for the EOLCI was 0.32, and for the Stanford model it was 0.48. This means that the binarized EOLCI achieved 32% of the performance of a perfect prediction model, compared to 48% for the Stanford model.
